# WHtR-BFP dual-dimensional synergy: innovative construction of a nine-grid model for precision obesity management

**DOI:** 10.3389/fnut.2026.1768569

**Published:** 2026-06-19

**Authors:** Lei Chen, Aichun Li, Wenhao Chen, Junlai Zhou

**Affiliations:** School of Physical Education, Hainan Normal University, Haikou, Hainan, China

**Keywords:** body fat percentage, nine-grid model, obesity, personalized obesity management, waist-to-height ratio

## Abstract

**Introduction:**

Current obesity assessment tools often capture either fat distribution or overall adiposity, limiting precise risk stratification and individualized management. To address this gap, we developed a dual-dimensional framework for obesity assessment and tiered intervention.

**Methods:**

Guided by Multidimensional Health Management Theory and the Stages of Change Theory, we constructed the waist-to-height ratio (WHtR)-body fat percentage (BFP) Dual-Dimensional Synergistic Nine-Grid Model. Using WHtR and BFP as orthogonal axes, the model establishes a 3 × 3 risk matrix linking phenotype classification with stage-matched behavioral intervention and tiered management.

**Results:**

The model defines nine obesity-related phenotypes and translates them into a five-tier management system (T1–T5). In a proof-of-concept application involving 50 university students, all participants were successfully classified into specific grid zones and intervention tiers, supporting the operational feasibility of the model. Compared with body mass index (BMI)-based categorization, the model provided greater phenotypic granularity and identified discordance between central adiposity and total body fat burden.

**Conclusion:**

The WHtR-BFP nine-grid model provides a novel conceptual framework for obesity management by integrating central fat distribution and total adiposity into a unified risk stratification approach. It offers a structured pathway for more individualized obesity care, with potential applicability in primary care and digital health settings. Further validation, subgroup calibration, longitudinal outcome studies, and health economic evaluation are needed before broader implementation.

## 1 Introduction

Obesity is not only a core risk factor for a range of chronic non-communicable diseases (NCDs) such as type 2 diabetes, cardiovascular disease, and certain types of cancer (1), but also imposes a substantial burden on global health systems and economies, including soaring direct healthcare costs and productivity losses (2, 3). Despite multiple interventions implemented by governments and public health organizations, the global obesity epidemic remains unchecked (4), revealing significant limitations in current prevention and control strategies. Faced with this formidable challenge, academia and policymakers increasingly recognize the imperative to shift from macro-level, broad-brush management models toward a new paradigm of individual-centered “Precision Governance.” The cornerstone of this transformation lies in the development of obesity risk assessment tools that are more precise, comprehensive, and clinically practical.

However, obesity assessment metrics widely used in current clinical practice predominantly exhibit “unidimensional” characteristics, and their inherent limitations have become a bottleneck constraining the refinement of obesity management. For instance, waist circumference (WC) and waist-to-hip ratio (WHR) rely on operator skill and standardized procedures, exhibit significant measurement variability, and lack globally unified standards, thereby compromising cross-population comparability (5, 6). Bioelectrical impedance analysis (BIA), while simple and non-invasive, is susceptible to interference from factors such as hydration status and electrolyte balance, and its accuracy is often questioned in severely obese individuals (7). Skinfold thickness measurement similarly faces limited adoption due to high technical demands on operators (8). Overall, these tools reflect only one aspect of adiposity—either fat distribution or total fat mass—and fail to simultaneously capture the two core dimensions that jointly drive metabolic risk: “total body fat load” and “abnormal fat distribution.” This limitation may lead to misjudgments of individual risk (9), highlighting the necessity of transitioning toward multidimensional assessment.

Current international mainstream guidelines for obesity prevention and treatment, such as those issued by the World Health Organization (WHO) (10) and the National Heart, Lung, and Blood Institute (NHLBI) (11), still rely heavily on body mass index (BMI) as the core diagnostic criterion. This reliance persists despite widespread recognition of BMI’s critical inability to distinguish between adipose and lean tissue or to account for fat distribution (12), leading to misclassifications in individuals with high muscle mass or sarcopenia. Although some guidelines incorporate waist circumference as a supplementary indicator for abdominal obesity (13), it is rarely organically integrated with BMI, resulting in a fragmented assessment process (14). Consequently, while scientific consensus emphasizes that obesity risk is co-determined by “quantity” (total body fat) and “location” (fat distribution) (15), existing assessment systems fail to provide a concise, integrated tool to synergistically evaluate these two dimensions (16). This research gap directly contributes to coarse risk stratification and a “one-size-fits-all” approach to intervention, thereby severely hindering the advancement of personalized obesity management.

To address these gaps, this study integrates Multidimensional Health Management Theory and the Stages of Change Theory to construct a dual-dimensional obesity management grid model (17, 18). Using waist-to-height ratio (WHtR) and body fat percentage (BFP) as core axes, the model establishes a 3 × 3 matrix that enables dynamic closed-loop management through data analysis, risk stratification, and precision intervention. This approach overcomes the limitations of single-indicator tools while balancing accuracy and practicality, thereby providing a clear pathway for advancing personalized obesity management.

## 2 Literature review and theoretical foundation

### 2.1 Historical evolution and critical review of obesity assessment tools

The trajectory of obesity assessment tools reflects a shift from qualitative description to quantitative precision. Prior to the standardization of weight measurement instrumentation, the assessment of obesity relied primarily on subjective visual observation. In the 1940s, driven by the need for health risk assessment, the insurance industry systematically introduced height-weight tables, thereby advancing obesity assessment into a preliminary stage of quantification (19). The core computational logic underlying these tables, specifically the ratio of weight to the square of height, was derived from the “Quetelet Index,” proposed by the Belgian statistician Adolphe Quetelet in the mid-19th century (20). In 1972, American physiologist Ancel Keys and colleagues demonstrated that this index was strongly associated with obesity-related health risks, such as hypertension and diabetes, through cross-population epidemiological studies. They subsequently formally designated it the body mass index (BMI) (21), establishing it as the most widely utilized obesity screening tool globally (22, 23). However, as research in obesity epidemiology has advanced, the intrinsic limitations of BMI have become increasingly apparent. Calculated solely as the ratio of weight to height, BMI fails to differentiate the proportional contributions of adipose tissue, muscle mass, and skeletal structure to total body weight. Furthermore, it does not incorporate fat distribution characteristics—a core variable influencing health outcomes. Consequently, this leads to frequent under-diagnosis of visceral obesity and misestimation of risks for cardiovascular disease and metabolic syndrome, particularly among athletes with high muscularity, the elderly with sarcopenia, and populations exhibiting ethnic disparities in body composition (24, 25).

In light of the intrinsic limitations of BMI, academic research and clinical practice have increasingly pivoted toward the assessment of central obesity as a more precise indicator of cardiometabolic risk. Consequently, a constellation of anthropometric indices has emerged, including WC, WHR, and WHtR. Evidence demonstrates that these metrics offer a superior evaluation of visceral adipose tissue accumulation, thereby providing enhanced predictive accuracy for metabolic disorders, cardiovascular diseases, and various malignancies (26, 27). Notably, WHtR standardizes the assessment of central obesity across individuals of varying statures by eliminating the confounding variable of height (28, 29). Crucially, Ashwell et al. (30) confirmed that WHtR possesses a significantly superior predictive capacity for intra-abdominal fat (*r* = 0.83) compared to BMI (*r* = 0.69). Building on this foundation, a systematic review by Browning et al. (31) not only validated the prognostic excellence of WHtR regarding diabetes and cardiovascular outcomes but also established 0.50 as a robust global risk boundary while advocating the concise public health message of keeping waist circumference to less than half of height. However, despite the advantages of these indices in characterizing abdominal adiposity, their accuracy is heavily contingent upon standardized measurement protocols. Furthermore, discrepancies regarding measurement sites and cut-off values across various studies and clinical guidelines pose significant challenges to the consistency and comparability of assessment results (32).

Propelled by the demand for precise quantification of body composition, technological advancements have driven the evolution of obesity assessment tools toward greater refinement and analytical resolution. BIA serves as a convenient modality for body composition quantification. By measuring differentials in electrical impedance to rapidly estimate BFP, BIA offers advantages such as operational simplicity and immediate results, making it suitable for preliminary screening in large-scale populations (33). However, the accuracy of this method is susceptible to interference from physiological variables, including hydration status and skin temperature (34, 35). In contrast, imaging modalities such as Dual-energy X-ray Absorptiometry (DXA), Computed Tomography (CT), and Magnetic Resonance Imaging (MRI) are universally acknowledged as the “gold standards” for body composition analysis, achieving a qualitative leap in assessment precision (36). DXA allows for the differentiation of fat mass, lean soft tissue, and bone mineral content. Furthermore, CT and MRI enable three-dimensional visualization and quantification of subcutaneous and visceral adipose tissue distribution, thereby providing a micro-anatomical basis for investigating the mechanisms underlying obesity (37). Nevertheless, the widespread application of these technologies as ubiquitous screening tools is constrained by factors such as prohibitive costs, potential radiation exposure, and operational complexity. Consequently, their use is primarily restricted to scientific research or specific clinical diagnostic scenarios. A comparison of common obesity assessment tools is presented in Table 1.

**TABLE 1:** Comparison of commonly used obesity assessment tools and indicators.

Measurement category	Measurement tool	Method description	Advantages	Limitations
Anthropometry	Body mass index (BMI)	Weight (kg)/height (m)^2^	Simple calculation; applicable to diverse populations; suitable for large-scale screening and assessment. It is the preferred method for assessing obesity and related health risks.	Ethnic differences exist; distinct standards are required for different races. BMI cannot quantify body fat percentage, fat distribution (e.g., visceral vs. subcutaneous fat), or degree of metabolic disorder. It also ignores the influence of muscle mass.
	Waist circumference (WC); Waist-to-hip ratio (WHR); Waist-to-height ratio (WHtR)	WC: typically measured using a soft tape at the midpoint between the lower costal margin and the iliac crest. WHR: WC/hip circumference (measured at the maximum extension of the buttocks). WHtR: WC/height (Ht).	Simple operation, low cost, and stronger correlation with Visceral Adipose Tissue (VAT). WC: no calculation required; suitable for rapid clinical screening. WHR: reflects fat distribution in both the abdomen and buttocks. WHtR: eliminates the influence of Ht on WC; the cut-off value (0.5) is applicable across different races, sexes, and ages, offering greater equity and comparability.	WC: diagnostic cut-off points vary by race, sex, and region; does not account for differences in Ht and body shape. WHR: hip circumference is an independent factor for metabolic diseases, so the WHR calculation may weaken WC’s core predictive role; lacks a universally accepted diagnostic threshold. WHtR: relies on the accuracy of the WC measurement.
	Body Roundness Index (BRI)	364.2–365.5 × √(1−(WC/(2π))^2^/(0.5 × Ht)^2^)	Compared to traditional BMI and WC, BRI better predicts body fat percentage and visceral fat content. It demonstrates a stronger association with health issues such as metabolic syndrome and cardiovascular risk.	Dependent on the accuracy of WC and Ht measurements. Optimal cut-off values for different racial, sex, and age groups have not yet been unified.
Device-based methods	Bioelectrical impedance analysis (BIA)	Estimates VAT by passing a weak electric current through the abdomen to measure impedance, combined with abdominal shape information. Uses dedicated bioelectrical impedance devices.	By combining impedance and abdominal morphological information, it can quantify VAT more comprehensively.	In individuals with high visceral fat levels, it may underestimate the degree of visceral fat, leading to significant bias.
	Dual-energy X-ray absorptiometry (DXA)	The body is scanned under a low-dose X-ray beam. Estimates fat, muscle, and bone mass based on differences in X-ray absorption rates of different tissues; can indirectly assess VAT.	VAT volume estimated by DXA correlates strongly with MRI results and is commonly used. It is a reference method for assessing abdominal obesity.	As a two-dimensional imaging technique, DXA cannot directly distinguish between VAT and subcutaneous adipose tissue.
	Computed tomography (CT)/magnetic resonance imaging (MRI)	CT: uses multi-angle X-ray scans to reconstruct abdominal cross-sectional images, allowing for precise calculation of VAT area or volume. MRI: uses magnetic fields and radio waves (without ionizing radiation) to generate detailed abdominal images for VAT quantification.	These are the reference standards for assessing abdominal obesity, capable of directly measuring VAT area or volume.	CT: involves radiation exposure. Both: high cost.

Based on data from Rubino et al. (12) and Table 1 in the study by Wang et al. (63).

**FIGURE 1:**
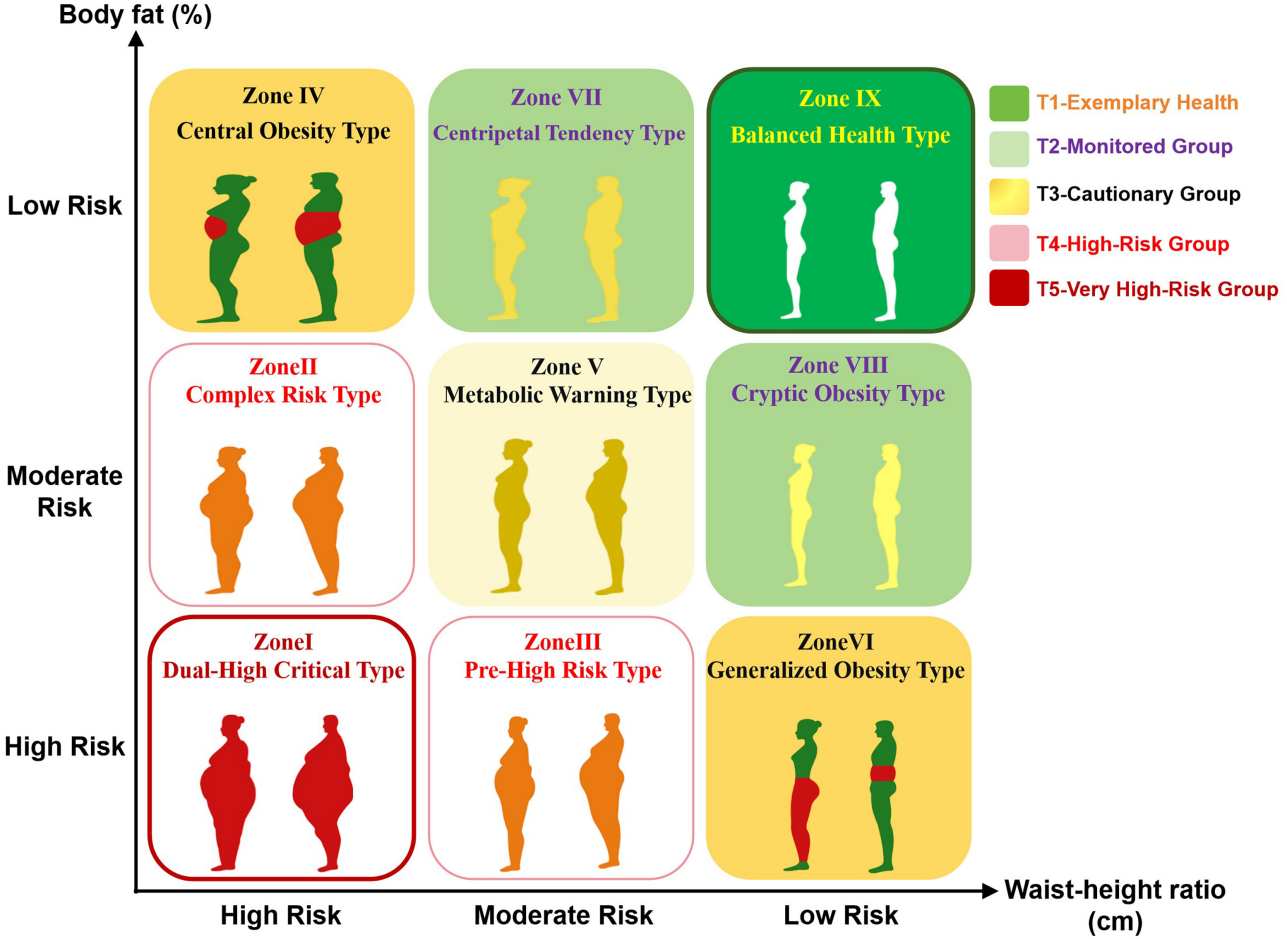
Waist-to-height ratio-body fat percentage dual-dimensional synergistic nine-grid model.

It is therefore evident that the critical bottleneck in current obesity assessment is not the lack of effective tools, but rather the absence of a systematic framework that can balance precision, practicality, and broad applicability. Furthermore, current obesity assessment lacks a hierarchical and complementary pathway that links broad preliminary screening with more precise individual-level diagnostic evaluation. To address this gap, we restructured the conventional obesity assessment framework by integrating WHtR and BFP as two complementary dimensions that capture central adiposity and overall body-fat burden, respectively. On this basis, we developed a nine-grid assessment model (Figure 1). Rather than relying on a single indicator, this model is designed to support dual-dimensional, integrated risk stratification and decision-making in obesity management.

### 2.2 Theoretical foundations for model construction

The construction of this model is predicated on the synergistic integration of the “Multidimensional Health Management Theory” with the “Stages of Change Theory.” The Multidimensional Health Management Theory provides a structural framework for analyzing obesity (38). It advocates transcending isolated physiological indices to systematically assess individual health across multiple dimensions, including physiological, psychological, interpersonal, societal, and environmental factors. This framework ensures the model can comprehensively identify the multifactorial determinants of obesity, thereby anchoring the direction for subsequent precision interventions. Complementarily, the Stages of Change Theory equips the model with a dynamic logic for behavioral regulation. This theory posits that health behavior change is a non-linear process progressing through five distinct stages: precontemplation, contemplation, preparation, action, and maintenance (39). Consequently, to maximize efficacy, management strategies should be tailored to the individual’s specific stage. For instance, interventions for individuals in the precontemplation stage, who may have limited awareness of obesity-related risks, should prioritize risk communication and awareness enhancement, whereas those in the preparation stage may require support in formulating concrete and feasible action plans.

**FIGURE 2:**
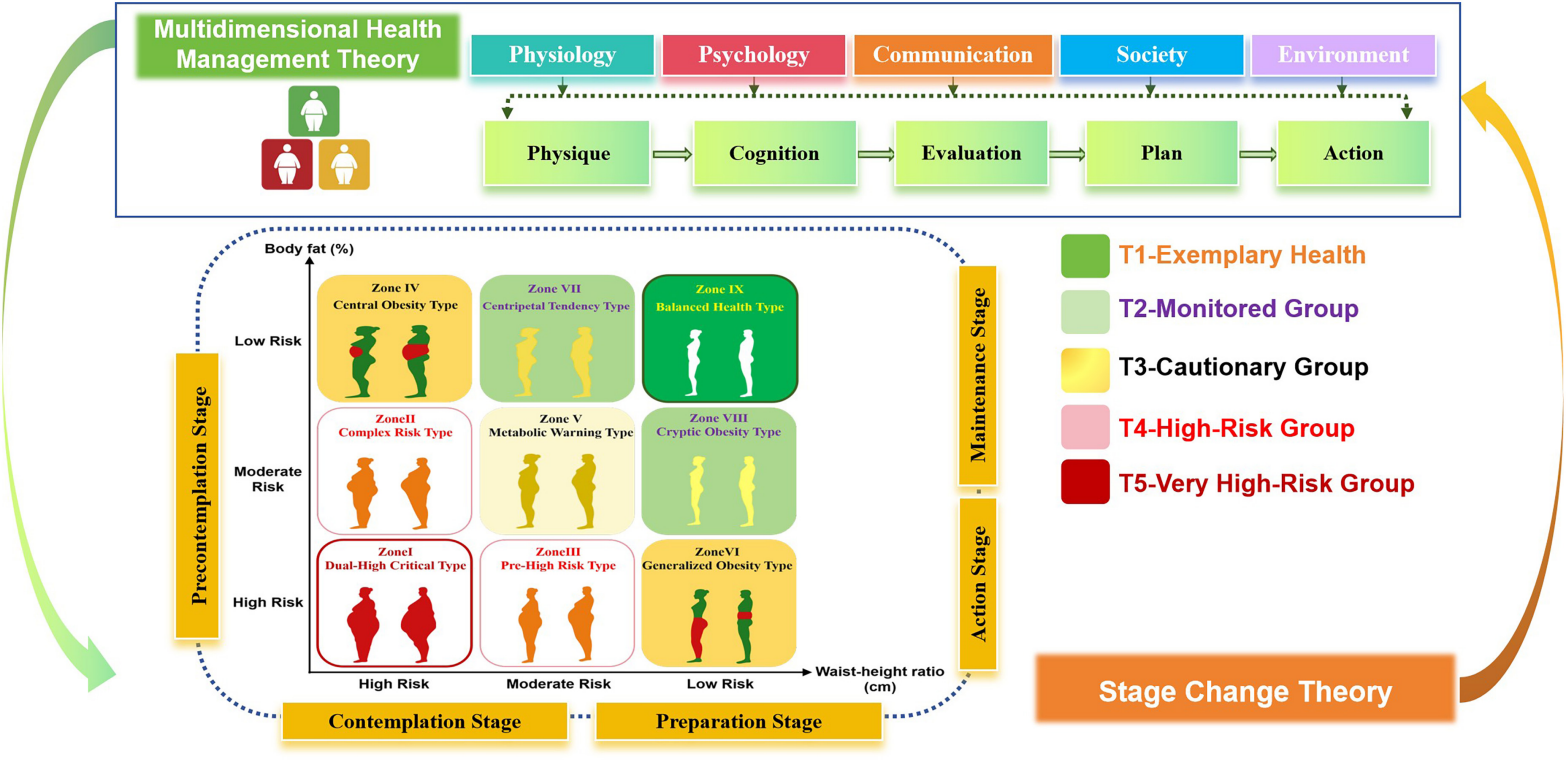
Theoretical framework of the waist-to-height ratio (WHtR)-body fat percentage (BFP) nine-grid model.

In summary, by innovatively synthesizing the “static structural dimension” of the former with the “dynamic process logic” of the latter, this model establishes a theoretical foundation for precision obesity management that is both systematic and developmental, as illustrated in Figure 2. This integration not only underpins the model’s capacity for a horizontal, multidimensional analysis of health issues but also provides the theoretical basis for guiding the vertical, progressive evolution of individual behaviors.

## 3 Innovative construction of the WHtR-BFP dual-dimensional collaborative nine-grid governance model

### 3.1 Construction of the assessment matrix and risk stratification

To operationalize the theoretical framework into a clinical tool, we constructed a dual-dimensional 3 × 3 matrix centered on risk stratification and dynamic tracking (Figure 1). The matrix operationalizes this dual-theoretical framework to facilitate quantitative analysis and iterative assessment-feedback-optimization cycles (40). Unlike unidimensional tools, this grid structure enables cross-classification of two obesity-related dimensions, generating distinct assessment units that can inform more tailored management strategies.

We selected WHtR and BFP as the orthogonal axes to capture the two core dimensions of obesity. WHtR was chosen to characterize central fat distribution and visceral metabolic risk (41), while BFP was selected to represent the total systemic fat burden (42). Crucially, these metrics demonstrate complementarity rather than collinearity (43), allowing the model to differentiate phenotypes often overlooked by single indicators, such as “non-obese visceral fat accumulation” (elevated WHtR with normal BFP) or “masked obesity” (elevated BFP with normal BMI) (44).

The model designates WHtR as the horizontal axis and BFP as the vertical axis. Each dimension is stratified into three risk tiers (low, moderate, and high) based on established clinical thresholds (Tables 2, 3), creating nine distinct stratification zones. These range from the “Balanced Healthy Type” (Zone IX: low/low) to the “Dual-High Critical Type” (Zone I: high/high). This 3 × 3 structure facilitates granular phenotype identification, effectively distinguishing between central obesity (high WHtR dominant) and generalized obesity (high BFP dominant), thereby providing a visual basis for precision intervention.

**TABLE 2:** WHtR risk classification criteria and definitions (unit: cm).

Risk level	Threshold	Definition	Physical characteristics
Low risk	<0.50	WC is less than 50% of Ht.	Less abdominal fat accumulation
Moderate risk	0.50–0.59	WC reaches or slightly exceeds 50% of Ht.	Signs of abdominal fat accumulation
High risk	≥0.60	WC exceeds 60% of Ht.	Pronounced central obesity

Integrated from the UK National Institute for Health and Care Excellence (NICE) guidelines (64) and the study by Ashwell and Gibson (65); WC, waist circumference; WHtR: waist-to-height ratio; Ht, height.

**TABLE 3:** Body fat percentage risk classification by region and population (unit: %).

Ethnicity/region	Low risk	Moderate risk	High risk	Core references
European/Caucasian	M: 8%-20%, F: 21%-32%	M: 21%–25%, F: 33%–38%	M: ≥26%, F: ≥39%	ACSM, ESPEN
Asian	M: 10%–18%, F: 20%–28%	M: 19%–23%, F: 29%–34%	M: ≥24%, F: ≥35%	IDF
South/Southeast Asian	M: 11%–19%, F: 22%–30%	M: 20%–24%, F: 31%–36%	M: ≥25%, F: ≥37%	IDF, SEARO
African (Sub-Saharan)	M: 12%–21%, F: 23%–33%	M: 22%–26%, F: 34%–39%	M: ≥27%, F: ≥40%	WHO Africa
Hispanic/Latin American	M: 13%–22%, F: 24%–34%	M: 23%–27%, F: 35%–40%	M: ≥28%, F: ≥41%	WHO

M, male; F, female; IDF, International Diabetes Federation (66); WHO, World Health Organization (67); AHA, American Heart Association (68); ACSM, American College of Sports Medicine (69); ESPEN, European Society for Clinical Nutrition and Metabolism (70).

### 3.2 Tiered configuration and management strategies

**FIGURE 3:**
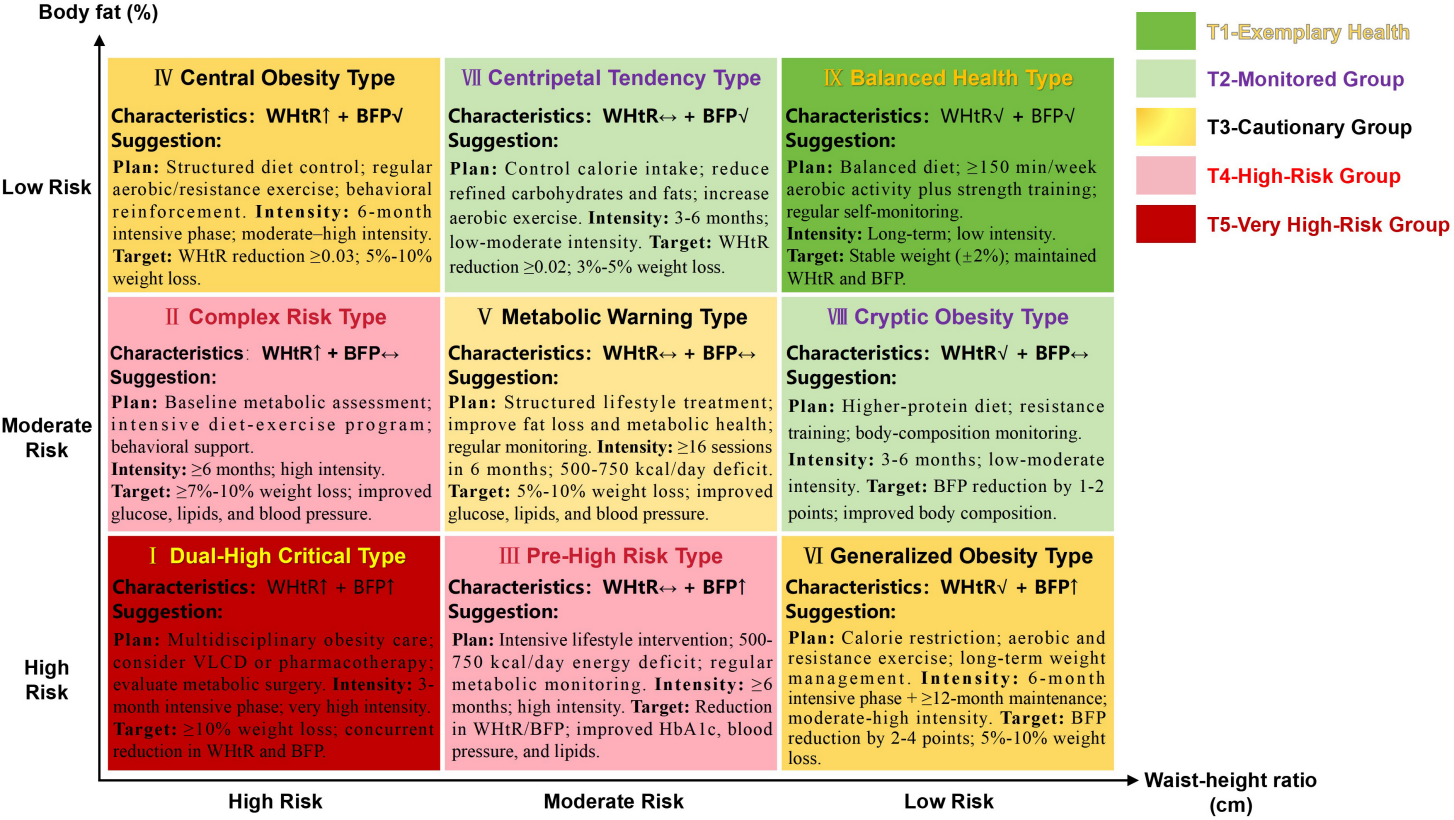
Tiered management strategy based on the waist-to-height ratio (WHtR)-body fat percentage (BFP) nine-grid model.

Drawing on the iCARDIO 2025 Guidelines (45) and the ADA Standards of Care in Diabetes (46), we translated the WHtR-BFP nine-grid assessment into a practical tiered management framework. Specifically, the target population was stratified into five intervention tiers (T1–T5) according to the intersection of WHtR- and BFP-defined risk levels. In this framework, WHtR reflects central adiposity and visceral cardiometabolic risk, whereas BFP reflects total body fat burden. Thus, tier assignment indicates not only the severity of adiposity abnormality but also the required intensity of management. To improve operational clarity and reproducibility, each tier was defined by its corresponding phenotype, intervention focus, duration/intensity, follow-up schedule, and measurable treatment targets. The concise operational definitions are presented in Figure 3, and the full tier-specific intervention specifications are provided in Supplementary Table 1.

During active management, anthropometric and clinical indicators should generally be reassessed at least every 3 months, whereas lower-risk maintenance states may be reviewed every 6–12 months. Across tiers, management intensity escalates progressively from health maintenance and targeted monitoring to structured lifestyle intervention, intensive non-pharmacological management, and multidisciplinary medical obesity care.

Tier 1 (T1, exemplary health) corresponds to Zone IX and represents a low-risk phenotype with both WHtR and BFP in the healthy range. Management is focused on prevention and long-term health maintenance through a balanced diet, regular physical activity, and routine self-monitoring, with the goal of maintaining stable body weight and adiposity status.

Tier 2 (T2, monitored group) includes Zone VII and Zone VIII and represents an early or discordant adiposity phenotype in which only one dimension is abnormal or borderline abnormal. Management remains targeted and phenotype-specific, with emphasis on central fat reduction in Zone VII and body-composition improvement in Zone VIII. The main goal is early correction before progression to more established obesity phenotypes.

Tier 3 (T3, cautionary group) includes Zone IV, Zone V, and Zone VI and represents established adiposity abnormality requiring structured multicomponent lifestyle treatment. This tier emphasizes integrated nutrition, exercise, and behavioral support, with the aim of achieving clinically meaningful fat loss, reduction in WHtR and/or BFP, and improvement in intermediate metabolic risk markers.

Tier 4 (T4, high-risk group) includes Zone II and Zone III and represents a high-risk combined phenotype with substantial adiposity abnormality and an increased likelihood of obesity-related metabolic complications. Intensive non-pharmacological intervention with medical oversight is required, including baseline metabolic evaluation, individualized calorie restriction, progressive exercise prescription, and behavioral support. The primary objective is to achieve substantial weight reduction and improvement in cardiometabolic risk indicators.

Tier 5 (T5, very high-risk group) corresponds to Zone I and represents the most severe phenotype, characterized by marked central adiposity together with excess total body fat. This tier requires comprehensive medical evaluation and multidisciplinary obesity management, with escalation to pharmacotherapy, medically supervised very-low-calorie diet, or metabolic surgery assessment when indicated. The main targets are major weight reduction, concurrent improvement in WHtR and BFP, and mitigation of obesity-related complications.

**FIGURE 4:**
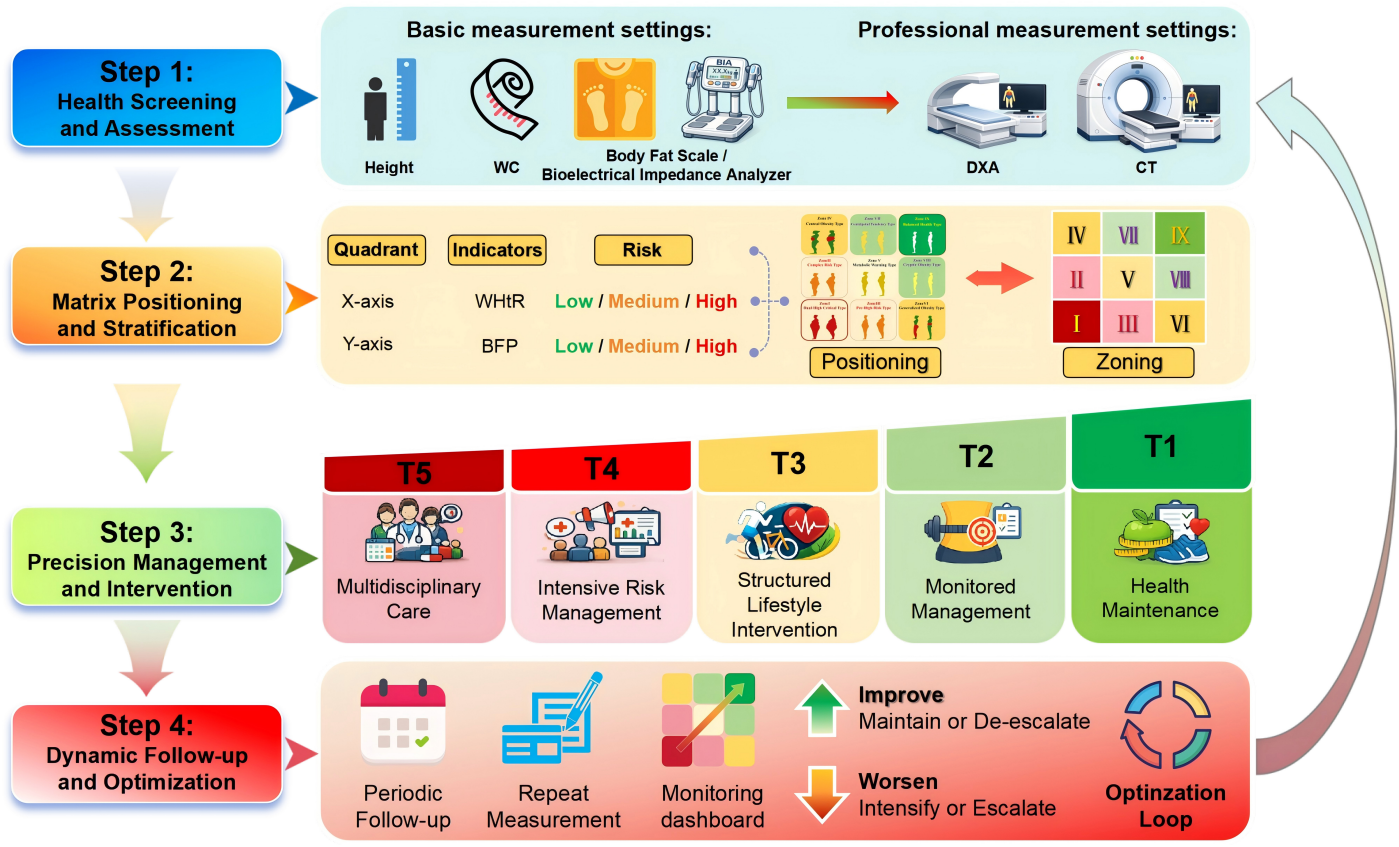
Workflow for the construction of the waist-to-height ratio (WHtR)-body fat percentage (BFP) dual-dimensional synergistic nine-grid model and its tiered management pathway.

Importantly, T1–T5 are intended not merely as descriptive risk labels, but as a tiered management framework linking phenotype classification to intervention intensity and objective treatment targets. To facilitate real-world implementation, we further translated this framework into a practical workflow (Figure 4), in which WHtR-BFP assessment is followed by nine-grid classification, tier assignment, phenotype-matched intervention, scheduled reassessment, and treatment escalation when response targets are not achieved.

### 3.3 Proof-of-concept evaluation in a pilot sample

To provide preliminary empirical support for the WHtR-BFP framework, we applied the model in a convenience sample of 50 university students. Height, weight, waist circumference, and body fat percentage were obtained using standardized field measurements, and all participants provided written informed consent before assessment. BMI was classified according to Chinese adult criteria (47), whereas WHtR and BFP were categorized using the predefined thresholds of the present nine-grid model.

**FIGURE 5:**
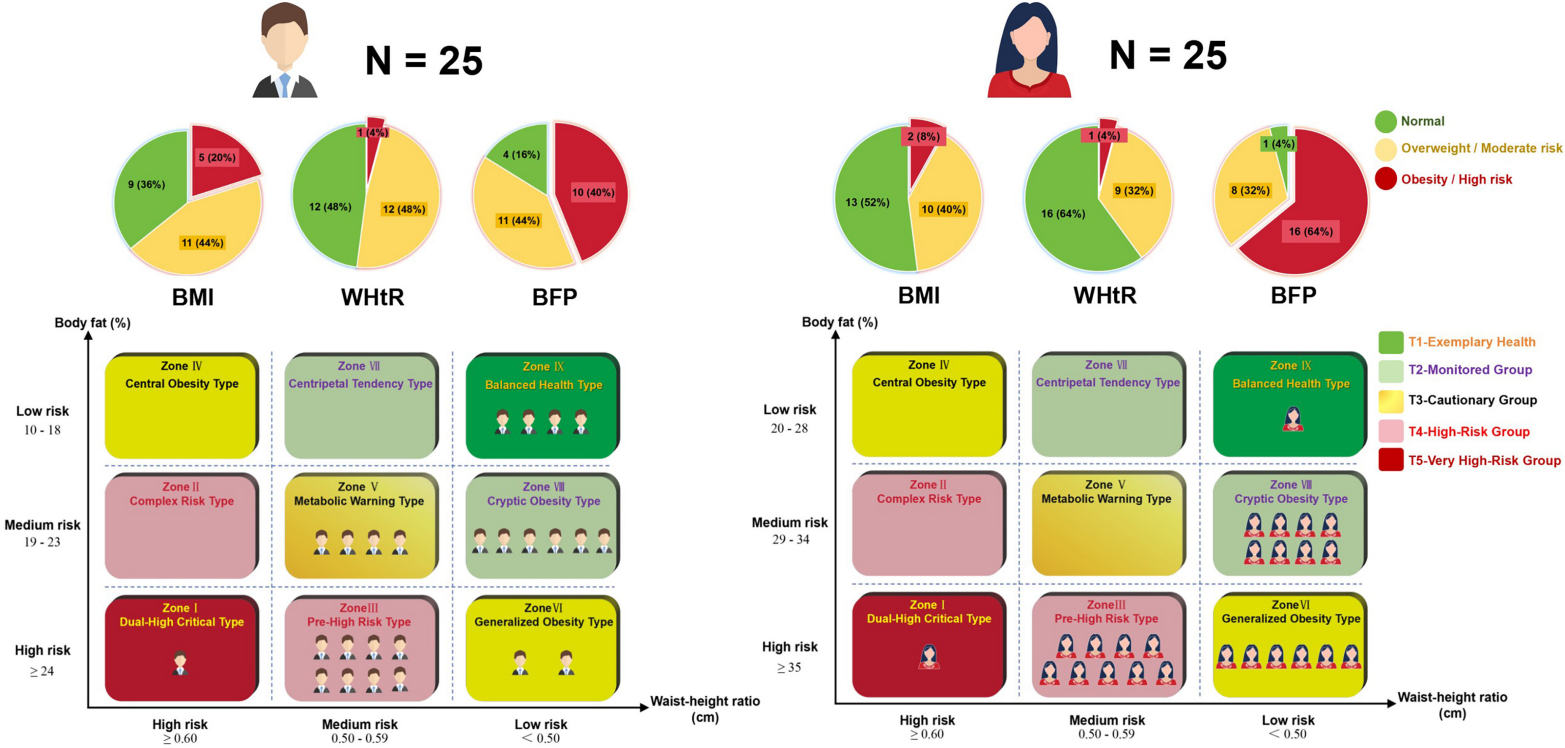
Pilot application results of the waist-to-height ratio (WHtR)-body fat percentage (BFP) dual-dimensional synergistic nine-grid model.

All 50 participants were successfully classified into specific WHtR-BFP zones and corresponding intervention tiers (T1–T5), supporting the operational feasibility of the framework (Figure 5). According to conventional BMI criteria, 22 participants were classified as normal weight, 21 as overweight, and seven as obese. In comparison, the two-dimensional nine-grid model generated a more detailed distribution across the five intervention tiers, with five participants in T1, 14 in T2, 12 in T3, 17 in T4, and 2 in T5. This distribution suggests that the proposed framework may offer greater phenotypic granularity than BMI-based categorization.

Sex-stratified analyses further revealed discordance between central adiposity and overall adiposity. Among male participants, WHtR-based risk levels were predominantly low to moderate (low, *n* = 12; moderate, *n* = 12; high, *n* = 1), whereas BFP-based risk classification indicated a higher level of overall fat accumulation (low, *n* = 4; moderate, *n* = 10; high, *n* = 11). A similar pattern was observed among female participants. Although low WHtR-based risk was common in females (*n* = 15), most were classified as having high BFP-based risk (*n* = 16). These results indicate that central adiposity and overall adiposity were not fully concordant in this sample, supporting the combined use of WHtR and BFP for risk stratification.

## 4 Discussion

This study innovatively constructs the WHtR-BFP dual-dimensional synergistic nine-grid model, shifting obesity management from single-indicator screening toward multidimensional precision care. By orthogonally integrating WHtR and BFP, the model establishes a dual-axis stratification framework that captures the interplay between fat distribution and total adiposity. The resulting clinical pathway—comprising data analysis, risk stratification, and precision intervention—helps address diagnostic blind spots inherent to unidimensional metrics. Ultimately, through its visually intuitive matrix structure, the model offers a scientifically robust and clinically actionable strategy to advance precision obesity management.

### 4.1 Decoupling risks for precision profiling

Current obesity screening strategies, including the WHO-endorsed combination of BMI and waist circumference (48), have advanced beyond unidimensional metrics but remain constrained by a static binary classification logic that merely aggregates independent cut-offs rather than capturing their synergistic effects on metabolic risk. Consequently, these methods often fail to reveal the complex interplay between fat distribution and systemic adiposity. To address this limitation, our model employs an orthogonal integration of WHtR and BFP which facilitates a true two-dimensional risk stratification. This approach is grounded in empirical evidence such as the findings of Ehrampoush et al. (49) showing that WHtR outperforms traditional indices like WHR in predicting body fat percentage and cardiometabolic risk. By establishing WHtR as the primary axis for central adiposity and coupling it directly with BFP, our framework operationalizes these insights to maximize diagnostic precision. Although emerging metrics such as the Body Roundness Index (BRI) show promise (50), WHtR may provide greater threshold stability across populations alongside the operational simplicity required for scalable screening (51). Ultimately, this matrix resolves vague obesity definitions into nine discrete physiological states and shifts intervention targets from generic weight loss toward more specific management of abdominal and systemic adiposity.

### 4.2 Coupling risk assessment with behavior change

The principal theoretical advancement of this study lies in its integration of static risk assessment with dynamic behavior change—a coupling mechanism that meaningfully distinguishes this model from conventional approaches. Traditional tools often function primarily as static risk labels and, rather than motivating action, may inadvertently provoke denial, disengagement, or resistance (52). In contrast, the proposed model establishes a set of structural coordinates that enable dynamic positioning of individuals along a behavioral continuum. By embedding the logic of the Stages of Change Theory (53), the model provides a form of temporal navigation by guiding individuals through stage-specific behavioral transitions as they move across risk zones. Accordingly, the T1–T5 tiers are not merely gradations of risk, but also mappings of behavioral readiness. This alignment resonates with Prochaska’s foundational insight that interventions mismatched to an individual’s stage of change are a primary driver of poor adherence (54). For example, individuals classified as T4 (High-Risk) are typically in the precontemplation stage, where the priority is raising awareness of health risk, rather than imposing immediate behavioral demands. This spatiotemporal integration also aligns with the logic of Just-in-Time Adaptive Interventions (JITAI) (55), forming a closed-loop control system that shifts the paradigm from disease-centered, episodic care toward person-centered, continuous dynamic regulation.

### 4.3 Allocating resources via stepped intervention

The stratified intervention strategy proposed here is consistent with the core tenets of the Stepped-Care Model, maximizing the allocative efficiency of medical resources through the principle of least intervention (56). As demonstrated by Bandurska et al. (57) in their study on pediatric obesity management in Poland, high-intensity multidisciplinary interventions are often limited in large-scale public health applications due to unfavorable cost-benefit ratios, while low-intensity alternatives may fail to address the pathological roots in high-risk populations. By translating abstract theory into an executable precision triage framework through the T1–T5 tiered system, the WHtR-BFP nine-grid model supports a continuum of care linking community-based prevention with hospital-based treatment. This bidirectional approach may help reduce the mismatch between excessive resource use in lower-risk groups and insufficient intervention in higher-risk individuals, while also offering a standardized, data-informed framework for integrated obesity management across healthcare settings. In this sense, the model does more than classify risk; it provides a practical basis for matching treatment intensity to clinical need, thereby supporting more coordinated escalation from health education and lifestyle counseling to multidisciplinary management and specialist referral.

### 4.4 Clinical applicability and population-specific considerations

From a public health and clinical management perspective, the WHtR-BFP nine-grid model may provide a scalable and resource-sensitive approach to obesity management across different levels of care. Rather than relying on uniform high-cost phenotyping, it could be applied pragmatically by combining routine anthropometric measures with accessible body composition assessment for initial classification in primary care and community settings, while reserving higher-precision techniques such as DXA or CT for specialist services, complex cases, or research settings. This flexibility may help preserve the clinical value of the model while improving feasibility in resource-limited environments. The structured, rule-based nature of the nine-grid matrix also facilitates integration into mobile health platforms and clinical decision-support systems, with potential utility for automated classification, longitudinal follow-up, and tier-matched intervention delivery. At the same time, broader implementation will require careful interpretation across populations. The current model is primarily applicable to adults, and its thresholds and clinical implications may need to be considered in relation to age, sex, and ethnicity. This is particularly important because body fat percentage and fat distribution vary across the life course, differ between men and women (58), and in women may also be influenced by menstrual status, reproductive stage, and menopause-related changes in body composition (59, 60). In addition, ethnic differences in adiposity and metabolic risk suggest that, although the WHtR-BFP nine-grid model may be broadly applicable, threshold calibration may still be needed for different populations (61, 62).

### 4.5 Limitations and future research directions

While the proposed WHtR-BFP nine-grid model provides a structured framework for precision obesity assessment and management, several limitations should be acknowledged. First, this study primarily presents a conceptual, review-based framework rather than a prognostic model derived from prospective clinical cohorts; therefore, the current model should be regarded as hypothesis-generating rather than fully validated for outcome prediction. In addition, its generalizability remains uncertain. At present, the model is intended primarily for adult populations, whereas its utility in children, adolescents, and older adults requires dedicated validation. Although sex-specific body fat ranges were considered, body composition may still vary according to sex-specific biological factors, and in women may be influenced by menstrual status and menopause, which were not explicitly modeled in the present framework. Likewise, ethnic heterogeneity should be taken into account, as both body fat percentage and obesity-related risk may differ across populations; thus, the proposed thresholds should not be considered universally fixed and may require calibration in diverse cohorts.

Second, measurement-related constraints may affect both feasibility and classification reliability. WHtR depends on standardized waist circumference measurement, and BFP may vary according to the method used. Although techniques such as DXA and CT can improve phenotypic precision, their limited accessibility reduces scalability, whereas more feasible approaches such as BIA may introduce method-related variability. Whether the model can retain adequate discriminative performance when supported by lower-cost and more accessible tools remains to be established. Finally, the framework has not yet undergone longitudinal outcome validation, and it remains unclear whether nine-grid classification predicts incident diabetes, cardiovascular events, or other clinically meaningful outcomes, or whether changes in grid position over time reflect true changes in health risk. Future research should therefore focus on multicenter validation in broader adult populations, subgroup analyses by age, sex, menopausal status, and ethnicity, comparisons across body composition assessment methods, and prospective studies evaluating both prognostic performance and clinical utility.

## 5 Conclusion

The WHtR-BFP dual-dimensional synergistic nine-grid model provides a novel conceptual framework for obesity management by integrating central fat distribution and total adiposity into a unified risk stratification approach. By coupling phenotype identification with stage-matched behavioral intervention, it transcends the limitations of unidimensional tools and establishes a structured pathway for individualized precision care. Its visual matrix structure and tiered protocols offer potential utility for community screening, primary care triage, and integration into digital health platforms. However, its broader clinical and public health implementation will depend on further multicenter validation, calibration across relevant population subgroups, longitudinal outcome studies, and health economic evaluation.

## Data Availability

The raw data supporting the conclusions of this article will be made available by the authors, without undue reservation.
