# Supplementary material for: WHtR-BFP dual-dimensional synergy: innovative construction of a nine-grid model for precision obesity management
Source: Front Nutr. 2026 Jun 19;13:1768569. doi: 10.3389/fnut.2026.1768569 (PMC13327881; doi:10.3389/fnut.2026.1768569)
Supplement: Supplementary file 1 [file Table_1.docx]

Supplementary Table 1 Full tier-specific intervention specifications for the WHtR-BFP dual-dimensional synergistic nine-grid model.

| **Tier** | **Corresponding zones** | **Operational definition** | **Intervention components** | **Duration and intensity** | **Follow-up and measurable outcomes** |
| --- | --- | --- | --- | --- | --- |
| **T1**  **(Exemplary Health)** | **Zone IX** | Low-risk phenotype with both WHtR and BFP within the low-risk range; no indication for active obesity treatment, but continued prevention is recommended. | General health education; balanced dietary pattern; ≥150 min/week of moderate-intensity aerobic activity plus resistance training 2-3 times/week; self-monitoring of body weight and waist circumference; positive reinforcement. | Ongoing, low-intensity management. | Reassessment every 6-12 months. Key targets: body weight maintained within ±2% of baseline, no increase in WHtR or BFP, and sustained adherence to ≥150 min/week of physical activity. |
| **T2**  **(Monitored Group)** | **Zone VII,**  **Zone VIII** | Early or discordant adiposity phenotype characterized by isolated or borderline abnormality in one dimension (central adiposity or systemic adiposity). | Phenotype-specific correction: for Zone VII, reduction of refined carbohydrates and energy-dense foods plus aerobic and core-focused exercise; for Zone VIII, reduction of fat mass and preservation of lean mass via higher-protein intake and resistance training. | Initial intervention for 3-6 months; low-to-moderate intensity. | Reassessment every 3 months. Key targets: WHtR reduction ≥0.02 or BFP reduction by 1-2 percentage points; body weight reduction of approximately 3-5%; establishment of regular exercise behavior. |
| **T3**  **(Cautionary Group)** | **Zone IV,**  **Zone V,**  **Zone VI** | Established obesity-related phenotype requiring structured multicomponent intervention; includes central obesity-dominant, generalized adiposity-dominant, or mixed intermediate-risk patterns. | Structured lifestyle intervention including nutrition, exercise, and behavioral counseling; ≥16 counseling sessions within 6 months; individualized dietary prescription targeting a 500-750 kcal/day energy deficit; phenotype-based emphasis on abdominal fat reduction, total fat reduction, or combined metabolic improvement. | Intensive phase of 6 months, followed by maintenance for ≥12 months; moderate-to-high intensity. | Follow-up every 1-3 months. Key targets: 5-10% weight loss; WHtR reduction ≥0.03 or reduction below the next risk boundary; BFP reduction by 2-4 percentage points; improvement in glycemia, blood pressure, triglycerides, or related metabolic indices. |
| **T4**  **(High-Risk Group)** | **Zone II,**  **Zone III** | High-risk combined phenotype with substantial abnormality in both dimensions, but not the most severe form; high likelihood of obesity-related metabolic complications. | Baseline medical evaluation, including blood pressure, glucose/HbA1c, lipid profile, liver and kidney function, and complication screening; intensive lifestyle intervention; cognitive-behavioral support; progressive exercise prescription; wearable-assisted monitoring when feasible; individualized caloric restriction with a 500-750 kcal/day deficit. | Intensive management for at least 6 months, followed by maintenance for ≥12 months; high intensity; consider pharmacological treatment when indicated. | Follow-up at least every 3 months. Key targets: ≥7-10% weight loss; WHtR reduction ≥0.03-0.05; clinically meaningful BFP decline; improvement in HbA1c, blood pressure, lipid profile, and other cardiometabolic risk markers. |
| **T5**  **(Very High-Risk Group)** | **Zone I** | Very high-risk phenotype characterized by simultaneous marked central adiposity and excess total body fat; strongest indication for multidisciplinary obesity care and escalation to medical therapy. | Multidisciplinary management involving physician, dietitian, exercise specialist, and behavioral or psychological support; medically supervised short-term VLCD (800-1,000 kcal/day) in selected individuals; low-intensity supervised physical activity; evaluation for anti-obesity pharmacotherapy, preferably GLP-1 receptor agonists or dual GIP/GLP-1 receptor agonists where appropriate; evaluation for metabolic surgery when indicated. | Initial intensive medical phase for ≥3 months; highest intensity; long-term maintenance for ≥12 months. | During pharmacotherapy, assess efficacy and safety monthly for the first 3 months and at least quarterly thereafter. Key targets: ≥10% weight loss, with >15% as a feasible goal in high responders; concurrent reduction in WHtR and BFP; improvement in obesity-related complications; escalation of treatment if early response is inadequate. |
| **WHtR**, waist-to-height ratio; **BFP**, body fat percentage; **HbA1c**, glycated hemoglobin; **VLCD**, very-low-calorie diet; **GLP-1**, glucagon-like peptide-1; **GIP**, glucose-dependent insulinotropic polypeptide; **ADA**, American Diabetes Association. | | | | | |

**Table notes:**

1. This table was developed with reference to the iCARDIO 2025 Guidelines ([1](#_ENREF_1)) and the ADA Standards of Care in Diabetes—2025 ([2](#_ENREF_2)), and was further adapted to fit the WHtR-BFP dual-dimensional synergistic nine-grid framework proposed in this study.
2. The intervention specifications summarized here should be interpreted as a proposed operational framework rather than as direct guideline recommendations.
3. Zone definitions are based on the WHtR-BFP dual-dimensional synergistic nine-grid model proposed in this study.
4. Intervention intensity and follow-up frequency should be individualized according to clinical status, comorbidities, treatment response, and local resource availability.
5. Pharmacotherapy and metabolic surgery should be considered in accordance with applicable clinical guidelines, regulatory approval, and patient-specific indications.
6. Outcome thresholds are proposed operational targets to support stratified management and should not be interpreted as fixed universal cutoffs for all populations.

# References:

1. Anker SD, Ji L, Kindel T, Coats AJ, Ojji D, Barragán AP, et al. iCARDIO Alliance Global Implementation Guidelines for the Management of Obesity: Focus on Prevention and Treatment of Cardiometabolic Disease. *J Assoc Physicians India* (2026) 74(3e):e1-e16. doi: 10.59556/japi.74.1445

2. American Diabetes Association Professional Practice Committee. 8. Obesity and weight management for the prevention and treatment of type 2 diabetes: Standards of Care in Diabetes-2025. *Diabetes Care* (2025) 48(Suppl 1):S167-80. doi: 10.2337/dc25-S008.
